# Photocatalytic Properties of Co_3_O_4_-Coated TiO_2_ Powders Prepared by Plasma-Enhanced Atomic Layer Deposition

**DOI:** 10.1186/s11671-017-2269-4

**Published:** 2017-08-16

**Authors:** Xi-Rui Zhao, Yan-Qiang Cao, Jun Chen, Lin Zhu, Xu Qian, Ai-Dong Li, Di Wu

**Affiliations:** National Laboratory of Solid State Microstructures and Department of Materials Science and Engineering, College of Engineering and Applied Sciences, Collaborative Innovation Center of Advanced Microstructures, Nanjing University, Nanjing, 210093 People’s Republic of China

**Keywords:** Plasma-enhanced atomic layer deposition, Commercial TiO_2_ powders (P25), Surface modification, Co_3_O_4_, Photocatalytic acitivity, P-n junction

## Abstract

Co_3_O_4_-coated commercial TiO_2_ powders (P25) p-n junction photocatalysts were prepared by plasma-enhanced atomic layer deposition (PEALD) technique. The structure, morphology, bandgap, and photocatalytic properties under ultraviolet light were investigated systematically. Although the deposition of Co_3_O_4_ does not change the anatase structure and crystallite size of P25 powders, the ultraviolet photocatalytic activity has been improved evidently. For the Co_3_O_4_-coated P25 powders, the trace Co ions exist as Co_3_O_4_ nanoparticles attached to TiO_2_ powder surface instead of the occupation of Ti^4+^ position in TiO_2_ lattice. The Co_3_O_4_-coated P25 powders exhibit enhanced photocatalytic degradation efficiency of almost 100% for methylene blue in 1.5 h under ultraviolet light, compared with P25 of 80%. The Mott-Schottky plots of photocatalyst powders confirm the p-n heterojunction formation in Co_3_O_4_–TiO_2_ nanocomposite materials, which is beneficial to increase the efficiency of photogenerated electron-hole separation. In addition, the Co_3_O_4_ coating also promotes the adsorption of organic dyes of methylene blue on P25 powders.

## Background

With the rapid development of modern industry, water contamination has emerged as a serious issue [1, 2]. Organic dyes present toxic effects and reduce light penetration in contaminated water [3]. Moreover, most textile dyes show recalcitrance towards chemical oxidation and other traditional wastewater treatment. Fortunately, TiO_2_-based photocatalysts exhibit excellent degradation towards organic dyes [4]. TiO_2_ has been extensively and intensively studied as one of popular photocatalytic materials due to its low toxicity, high chemical stability, and catalytic activity in elimination of a large range of organic pollutants [5–7]. However, its overall quantum efficiency is relatively low due to the fast recombination rate of photogenerated electron-hole pairs [8]. Besides, the intrinsic large band gap of TiO_2_ limits its optical absorption to the UV region, which only accounts for less than 4% of the total solar radiation [9, 10]. These flaws impede its practical applications. Hence, different approaches have been explored to improve its photocatalytic activities, including metal/nonmetal doping [11, 12], dye sensitization [13], and heterojunction formation [14, 15].

It has been demonstrated that building p-n hetero-junction between n-type TiO_2_ and p-type semiconductor, such as NiO or Ag_2_O, is beneficial for reducing the recombination rate of photogenerated electrons and holes [16–18]. Firstly, the p-n junction can produce a built-in potential at the semiconductor interface. Under illumination, the inner electric field will promote the separation and transportation of photogenerated electron-hole pairs [19]. Secondly, semiconductors with smaller bandgap can enhance the light absorption of catalyst with larger bandgap [20]. Moreover, some semiconductors can also be employed to improve the stability of catalyst and facilitate the surface electrochemical reactions [21]. As a result, the photocatalytic activity could be improved dramatically by the formation of semiconductor/semiconductor hetero-junction. Chen et al. have reported that p-n junction NiO/TiO_2_ photocatalyst showed improved photoactivity in degrading methylene blue (MB) [22].

Co_3_O_4_, one of the most versatile transition-metal oxides, is widely applied in many fields, such as dyes degradation [23, 24], gas sensors [25], lithium ion batteries [26], oxidation of CO at low temperature [27], and H_2_ generation [28]. Co_3_O_4_, like NiO and Ag_2_O, belongs to p-type semiconductors. Its bandgap (2.1 eV) is relatively narrower compared with that of NiO (3.5 eV). Moreover, it shows better chemical stability than Ag_2_O because Ag_2_O tends to absorb CO_2_ in air to form Ag_2_CO_3_ or decomposes into Ag when used at a comparatively high temperature [28]. It has been reported that p-n Co_3_O_4_/BiVO_4_ or Co_3_O_4_/TiO_2_ junction exhibited higher photocatalytic activity than single semiconductor of BiVO_4_ or TiO_2_ in removing organic dyes [29, 30].

Quite a few methods have been used to synthesize Co_3_O_4_-based nanosystems, such as chemical vapor deposition (CVD) [31–33], plasma spray [34], and plasma-assisted CVD (PECVD) processes [35–37]. The Co_3_O_4_/TiO_2_ p-n junction has also been fabricated by impregnating-deposition-decomposition method [30]. The subsequent calcination and excitation were needed, which might produce exhaust emission.

Atomic layer deposition (ALD) is a novel thin film deposition technique based on sequential self-limited and complementary surface chemisorption reactions using precursor vapor. Compared to CVD, PECVD, and chemical solution method, it exhibits unique advantages, including large area uniformity, excellent three-dimensional conformality, precise and simple control of film-thickness, flexible surface modification, and low processing temperature [38]. Plasma-enhanced atomic layer deposition (PEALD), where plasma species are employed as reactive gas during one step of the cyclic deposition process, shows some merits over thermal ALD, such as more freedom to the substrate temperature and precursors. Recently, ALD has shown increasing prospects and wide applications in various fields such as semiconductor [39], new energy [40], and photocatalysis [41], especially in the surface modification of nanomaterials [42].

Herein, trace Co_3_O_4_-coated TiO_2_ p-n junction photocatalyst was fabricated by ALD method. Compared with the impregnating-deposition-decomposition method with multi-step procedures [30], ALD technique has only one-step deposition and low processing temperature of 200 °C without subsequent annealing. The crystal structure, morphology, composition, and bandgap of Co_3_O_4_-coated P25 powders were characterized by various analytical techniques. The photocatalytic activity of Co_3_O_4_-coated P25 powders with 100 and 200 cycles in the degradation of methylene blue (MB) dye under ultraviolet (UV) light irradiation has been investigated deeply. It can be found that, in contrast to the pure P25 powders, the 100-cycle Co_3_O_4_-coated P25 p-n junction sample exhibits distinctly enhanced UV photocatalytic efficiency. The possible photocatalytic mechanism of Co_3_O_4_-coated TiO_2_ powders is also proposed.

## Methods

Commercial TiO_2_ powders (P25) were used as supporters for Co_3_O_4_ deposition. P25 powders were loaded uniformly into a porous container and placed in the PEALD chamber (SUNALE R-200, Picosun). Dicarbonyl cyclopentadienyl cobalt (CoCp(CO)_2_, Strem Chemicals, 96%) kept at 45 °C and room-temperature oxygen plasma was used as cobalt precursor and oxygen source for Co_3_O_4_ deposition, respectively. High purity oxygen (99.999%) was used as oxygen plasma source with argon (99.999%) as carrier gas, and the plasma power and O_2_ gas flow rate were 2500 W and 160 sccm, respectively. Then 100- and 200-cycle Co_3_O_4_ were deposited on P25 powders at 200 °C by PEALD, where one cycle consisted of 0.2 s CoCp(CO)_2_ dosing, 6 s N_2_ purging, 21.5 s O_2_ plasma dosing, and 6 s N_2_ purging. For the 600-cycle Co_3_O_4_-coated P25 sample, flowing oxygen (130 sccm) instead of oxygen plasma was used as oxygen source. The Co precursor and reactor temperature remained unchanged. Therefore, 600-cycle Co_3_O_4_ were deposited on P25 powders by thermal ALD, where one cycle consisted of 2 s CoCp(CO)_2_ dosing, 8 s N_2_ purging, 5 s O_2_ dosing, and 10 s N_2_ purging. In our previous work, it has been demonstrated that PEALD Co_3_O_4_ on carbon nanotubes showed a low deposition rate and island growth mode [43]. The thickness of 800- and 2400-cycle Co_3_O_4_ was 5 and 20 nm, respectively. The rough deposition surface was covered by Co_3_O_4_ nanoparticles. Therefore, 100- and 200- cycle Co_3_O_4_ deposition on P25 may be still in its nucleation stage, might leading to the formation of Co_3_O_4_ nanoparticles coated TiO_2_ p-n junction structure.

The crystal structure of Co_3_O_4_-coated P25 powders was characterized by X-ray diffraction (XRD, Rigaku-D/max 2000) with Cu Kα radiation (λ = 0.15418 nm). The scanning angle ranged from 10° to 80° operated at 40 kV and 40 mA. The surface chemical feature was analyzed via X-ray photoelectron spectroscopy (XPS, Thermo ESCALAB-Thermo fisher K-alpha) using Al Kα radiation (1486.6 eV) as the excitation source. All binding energies were referenced to the C 1s peak at 284.6 eV. Inductively coupled plasma mass spectrometry (ICP-MS, Thermo X Series 2 ICP-MS) was carried out to measure the Co element content of photocatalyst powders.

The microstructure and surface morphology of the powders were characterized using field-emission scanning electron microscopy (FESEM, Ultra 55, ZRISS) and transmission electron microscopy (TEM, FEI Tecnai G^2^ F20 S-Twin). The catalyst powders were dispersed fully in ethanol by 20 min ultrasonic vibration before dripping onto the copper grid with ultrathin carbon foil for TEM observation. The Brunauer-Emmett-Teller (BET) specific surface areas were carried out using nitrogen adsorption apparatus (Micromeritics Tristar-3000).

The photocatalytic activity of Co_3_O_4_-coated TiO_2_ powders in the decomposition of methylene blue (MB) was evaluated under irradiation of a 100-W UV LED lamp (UVEC-411). Circulating cooling water was employed to maintain the system temperature at ~ 25 °C. The lamp was located at 15 cm away from the reaction solution. Fifty milligrams of catalyst was added into 50 mL MB aqueous solution (37.4 mg/L). Prior to the illumination, the mixed solution was stirred for 3 h in the absence of light to achieve the adsorption equilibrium. After each given irradiation time, about 4 mL of the mixture was withdrawn and separated by centrifuging to remove the suspended solid catalyst. The degradation process was monitored by a UV-vis absorption spectrum (UV-3600, Shimadzu, Japan), and the concentration of the residual MB was analyzed quantitatively by measuring the maximum absorption at 664 nm.

The visible-light photocatalytic activity of Co_3_O_4_-coated TiO_2_ powders was also evaluated via the degradation of methyl orange (MO) in aqueous solution. A solar simulator (300 W Xe lamp, MircoSolar300, PerfectLight) with a 420-nm cut-off filter provides the visible-light irradiation. The concentration of residual MO was determined by measuring the maximum absorption of MO at 464 nm.

Mott-Schottky plots were measured using electrochemical working station (CHI Instruments CHI760E) at frequencies of 1 and 2 kHz in dark. Fifty-two-milligram P25 or 200-cycle Co_3_O_4_-coated P25 powders along with 18 mg iodine were dispersed in 50 mL acetone via ultrasonic vibration. Then, the mixed slurry was electroplated onto fluorine-doped tin oxide (FTO) conducting glass under 15 V for 2 min. The electrochemical measurement was conducted in 1 M NaOH electrolyte at room temperature using a three-electrode configuration. The as-prepared FTO glass with photocatalyst was adopted as the working electrode. A platinum mesh (1 cm × 2 cm) and Ag/AgCl were used as counter electrode and reference electrode, respectively. The isoelectric point (IEP) of MB, P25, and 200-cycle Co_3_O_4_-coated P25 in aqueous solutions was determined using the Zeta potential measurement (Malvern Zetasizer, Nano ZS 90 zeta).

## Results and Discussion

**Fig. 1:**
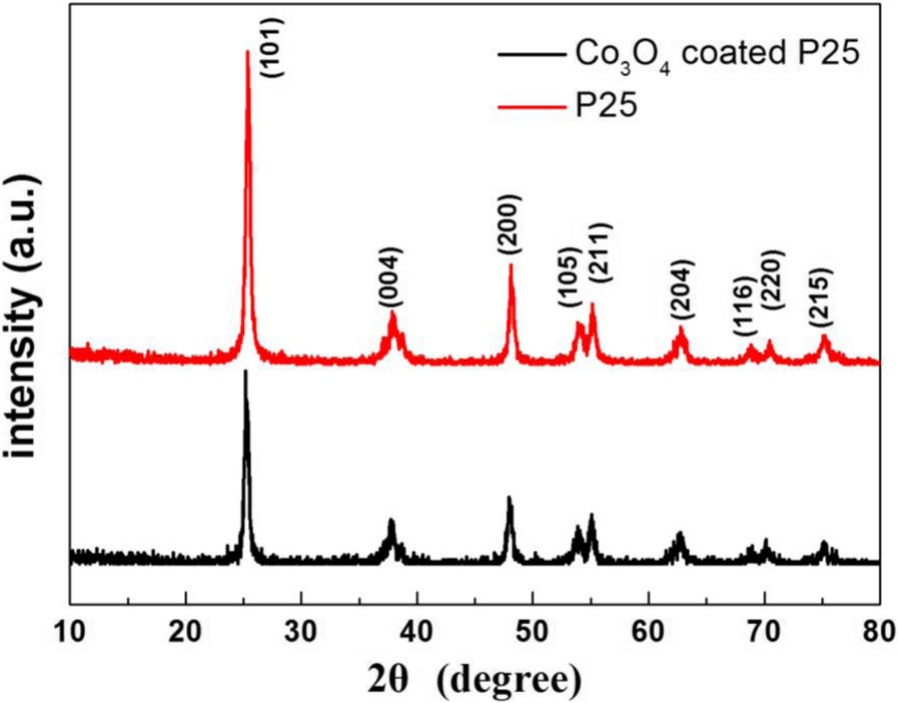
XRD patterns of pure P25 and 200-cycle Co_3_O_4_-coated P25 powders

XRD was used to determine the phase structure of the samples. Figure 1 exhibits the XRD patterns of pure P25 and 200-cycle Co_3_O_4_-coated P25 powders. Both samples display the similar characteristic peaks of standard anatase TiO_2_ (JCPDS card no: 21–1272), suggesting that there is no obvious change in the crystal structure after Co_3_O_4_ coating. In addition, the crystallite size of both samples can be estimated to be 20 ± 2 nm by Scherrer formula.

**Fig. 2:**
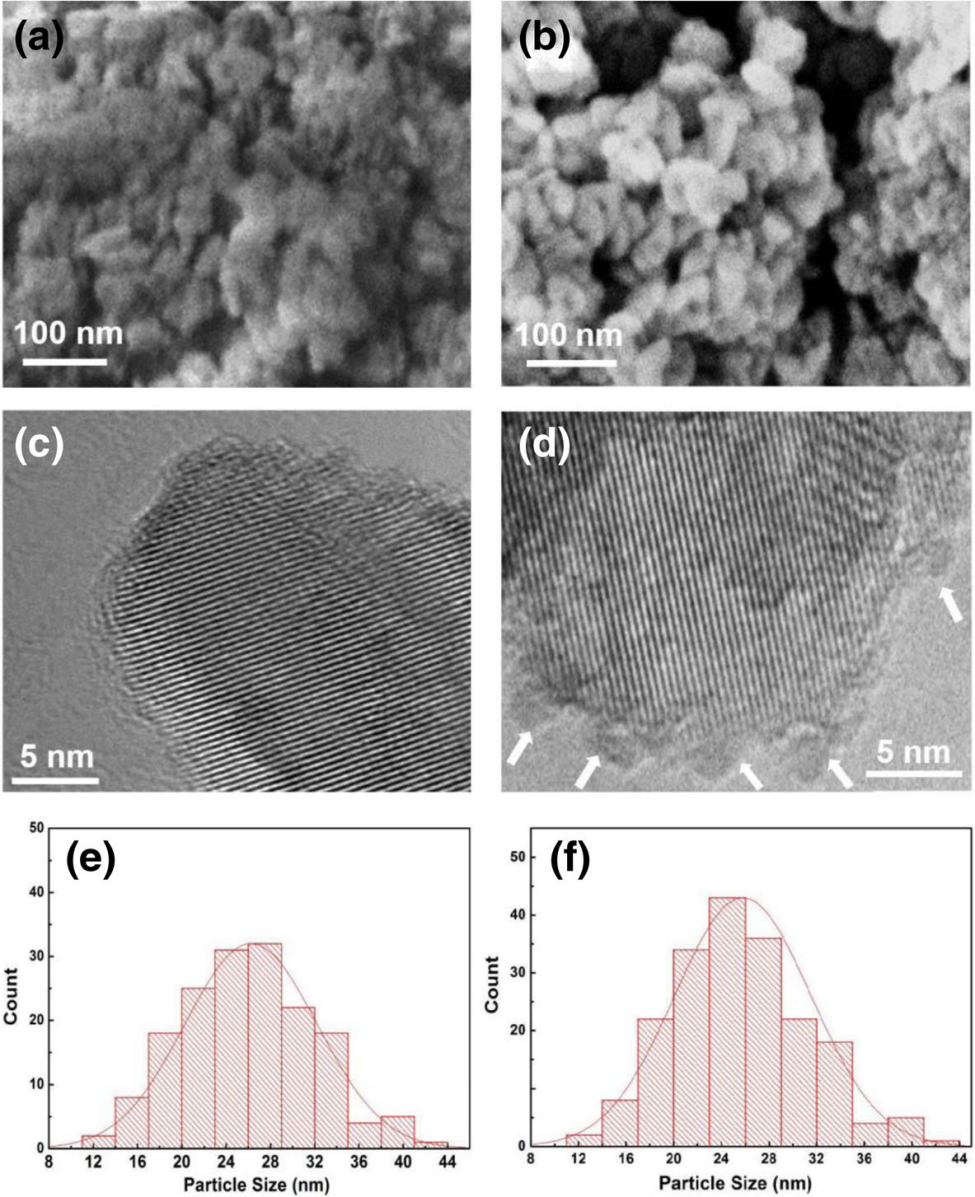
SEM (**a**, **b**) and HRTEM (**c**, **d**) images of pure P25 and 200-cycle Co_3_O_4_-coated P25 powders along with the particle size distribution (**e**, **f**). **a**, **c**, **e** Pure P25 powders. **b**, **d**, **f** 200-cycle Co_3_O_4_-coated P25 powders

SEM and TEM were utilized to observe the morphology and microstructure of pure P25 and 200-cycle Co_3_O_4_-coated P25 powders, as shown in Fig. 2a–d. Pure P25 and 200-cycle Co_3_O_4_-coated P25 samples show similar morphology and crystallite size of 15–30 nm (Fig. 2a, b). The nanoparticle size distribution was also counted, as shown in Fig. 2e, f, which can be fitted to Gaussian curves. The calculated average value of crystallite sizes of pure P25 and 200-cycle Co_3_O_4_-coated P25 powders is ~ 25.8 and ~ 26.2 nm, respectively, which is slightly larger than the XRD result due to the easy negligence of smaller nanoparticles in SEM observations. These nanoparticles agglomerate together to form some larger clusters of 50–100 nm. In high-resolution TEM (HRTEM) image of Fig. 2c, a local magnification well-crystallized TiO_2_ nanocrystal with clear lattice fringes can be seen in pure P25 powders. After 200-cycle PEALD Co_3_O_4_, we can notably discern some small amorphous nanoparticles located on the larger crystalline TiO_2_ surface with the diameter of 2–3 nm, as marked by arrows in Fig. 2d. Based on our previous work [43], these small nanoparticles should be the PEALD-derived Co_3_O_4_ with island growth mode. Combined with TEM and XRD results, it can be deduced that the Co ions exist as Co_3_O_4_ amorphous nanoparticles attached to TiO_2_ powder surface instead of the occupation of Ti^4+^ position in TiO_2_ lattice.

In addition, the influence of PEALD Co_3_O_4_ on the specific surface area of P25 was also examined. The BET surface area is 112.6 and 104.0 m^2^/g for pure P25 and Co_3_O_4_-coated P25 powders, respectively, so Co_3_O_4_ deposition on P25 powders has slight effect on the specific surface area of P25.

**Fig. 3:**
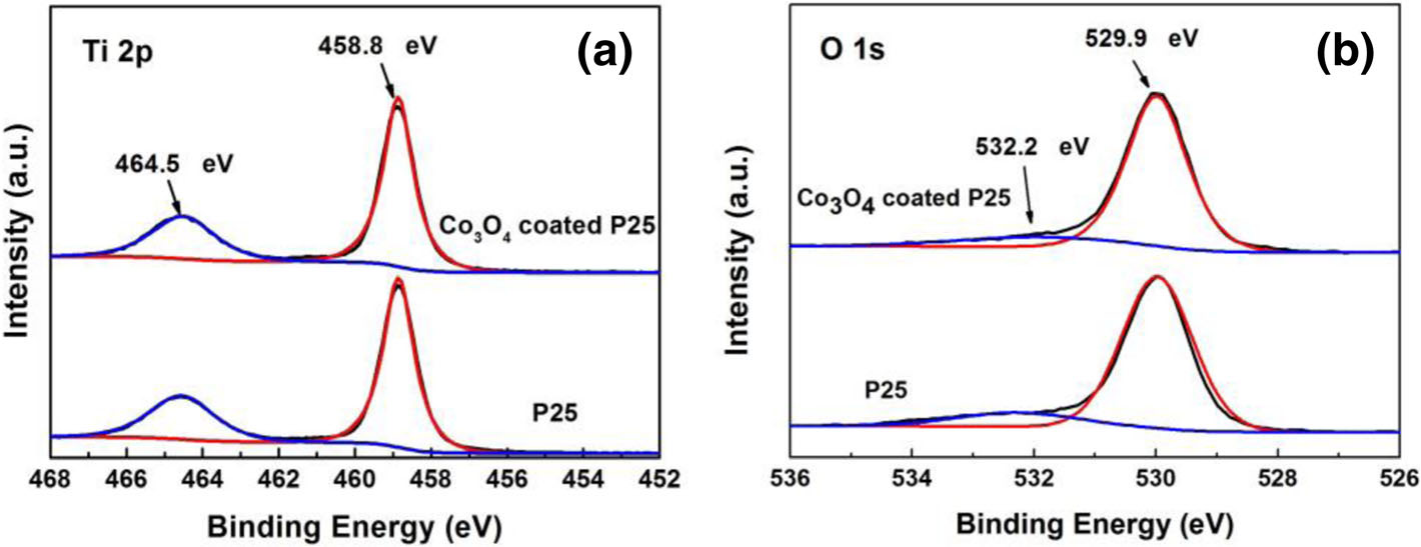
XPS spectra of 100-cycle Co_3_O_4_-coated P25 powders and pure P25 powders. **a** Ti 2p. **b** O 1s

XPS was performed to investigate the chemical composition of the samples with and without PEALD 100-cycle Co_3_O_4_ coating. Both samples show nearly the same signals for Ti 2p and O 1s spectra. In Fig. 3a, the doublet at 464.6 and 458.9 eV can be assigned to Ti^4+^ 2p_1/2_ and Ti^4+^ 2p_3/2_ peaks of Ti–O bonds with the spin orbit splitting energy of 5.7 eV, consistent with the values of TiO_2_. The O 1s spectra can be deconvoluted into two peaks, as shown in Fig. 3b. The strong peak at 529.9 eV can be assigned to the O–Ti bond. The weak peak with higher binding energy at 532.2 eV is attributed to the absorbed OH species on the sample surfaces [44]. The O1s peak of Co_3_O_4_ should locate at ~ 529.8 eV [43], which is difficult to be distinguished from the O–Ti bond. The calculated atomic ratio of Ti:O is about 1.00: 2.13, basically consistent with the composition of TiO_2_. However, the Co signal of 100-cycle Co_3_O_4_-coated P25 powders is too weak to be detected. It can be ascribed to the fact that Co content may be below the detection limit of XPS. Therefore, ICP-MS was utilized to determine the Co content in pure P25 and 100-cycle Co_3_O_4_-coated P25 powders. It is found that Co content in pure P25 and 100-cycle Co_3_O_4_-coated P25 is 0.13 and 3.63 ppm, respectively. Hence, trace Co_3_O_4_ is indeed deposited on the P25 powders by PEALD. In addition, XPS was also used to analyze 600-cycle Co_3_O_4_-coated P25 samples prepared by thermal ALD. The weak Co 2p spectra can be recognized with the Co atomic percentage content of ~ 0.6%.

**Fig. 4:**
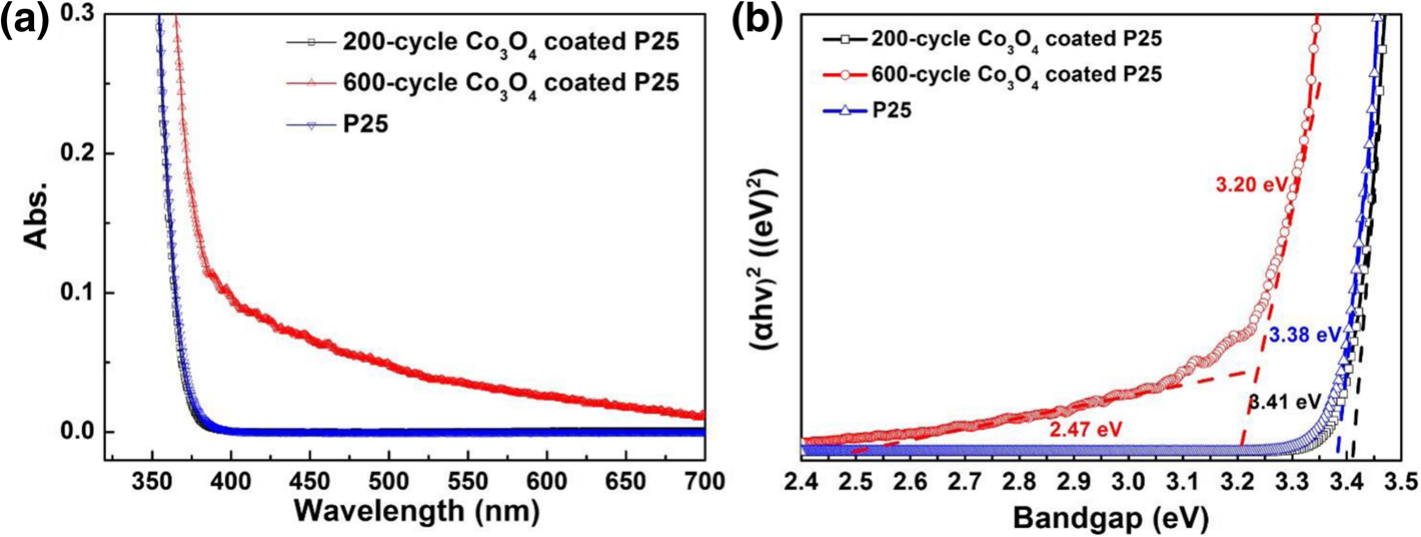
**a** UV-visible diffuse reflection spectra and (**b**) corresponding band gaps determination plots of pure P25, 200-cycle and 600-cycle Co_3_O_4_-coated P25 powders

Figure 4a records the room-temperature UV-visible diffuse reflection spectra of pure P25 and Co_3_O_4_-coated P25 samples with 200 and 600 cycles. Pure P25 and 200-cycle Co_3_O_4_-coated P25 samples show almost the same optical absorption spectra, however, 600-cycle Co_3_O_4_-coated P25 samples derived from thermal ALD exhibit relatively stronger absorption in the visible range from 400 to 700 nm, especially in 400–500 nm region, which originates from the d-d transition for Co^3+^ or Co^2+^ ions.

For the direct bandgap semiconductor, the relation between the absorption edge and the photon energy (*hν*) can be written as follows [45]:

(*αhν*)^2^ = *A*(*hν* − *E*_*g*_) where *A* is the absorption constant of the direct band gap semiconductor. The absorption coefficient (α) is determined from the scattering and reflectance spectra according to Kubelka-Munk theory. The direct bandgap energies can be estimated from the intercept of the tangents to the plots, as presented in Fig. 4b. The bandgap of 200-cycle Co_3_O_4_-coated P25 powders is about 3.41 ± 0.02 eV, almost the same as pure TiO_2_ powders (3.38 ± 0.02 eV), due to extremely low Co loading amount (~ppm by ICP-MS). Six hundred-cycle Co_3_O_4_-coated P25 samples show two bandgaps because of relatively higher Co loading (~ 0.6 atomic % by XPS). The larger bandgap of 3.20 ± 0.03 eV comes from TiO_2_ powders, whereas the much smaller bandgap of 2.47 ± 0.03 eV might be related to Co_3_O_4_ coating. ALD-derived Co_3_O_4_ coating has slightly wider bandgap than the literature value of 2.3 eV from Co_3_O_4_ nanospheres (~ 20 nm) by solution-based synthesis [46].

**Fig. 5:**
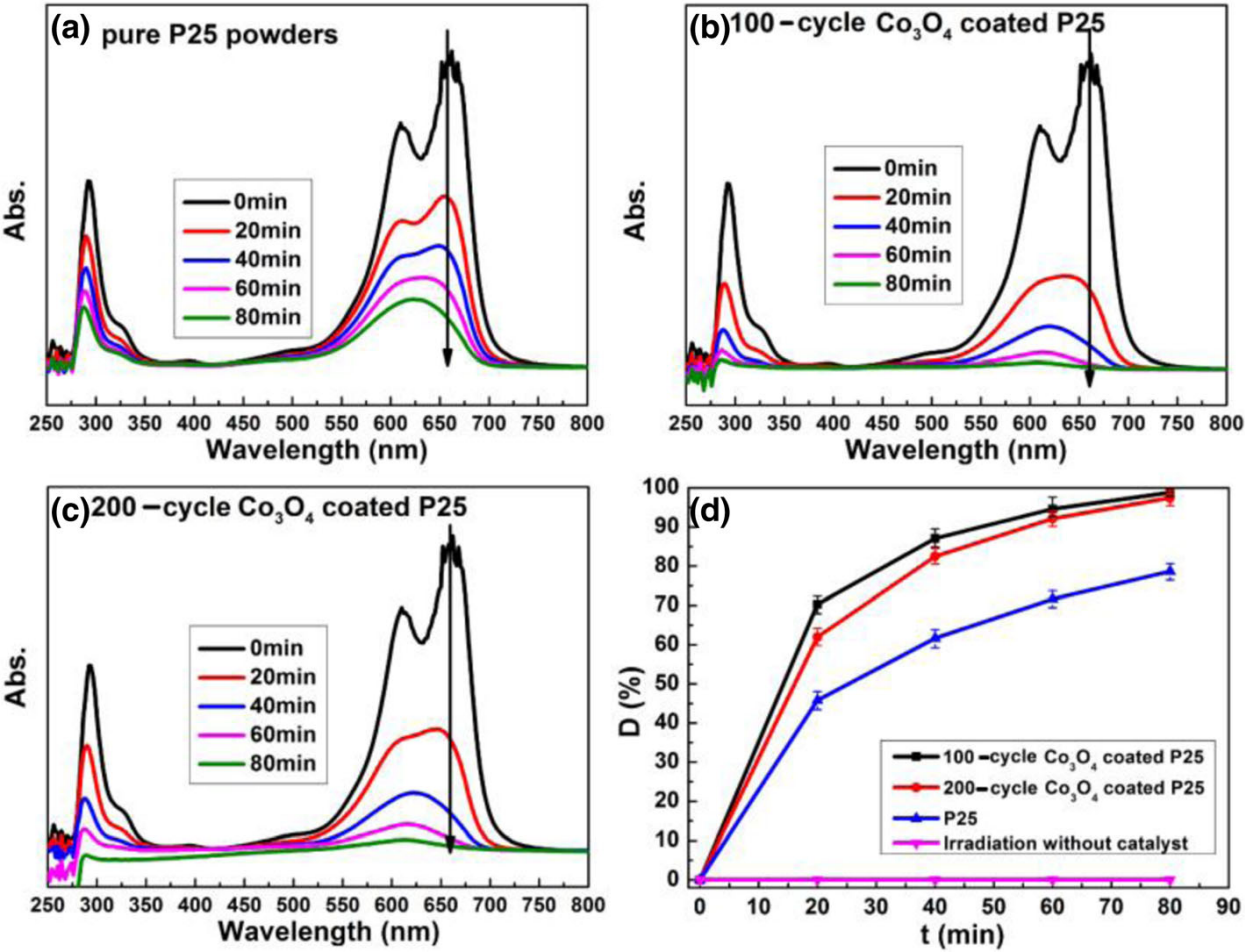
UV-visible absorption and degradation curves of MB solution with various catalysts. **a** Pure P25. **b** 100-cycle Co_3_O_4_. **c** 200-cycle Co_3_O_4_-coated P25 catalyst. **d** Degradation curves of MB

MB is commonly used as the probe for evaluating photocatalysts, and its degradation mechanism has been well clarified. Figure 5a–c illustrates the photocatalytic decomposition of MB under UV light in the presence of pure P25, 100-cycle, and 200-cycle Co_3_O_4_-coated P25 photocatalysts, respectively. The maximum absorption of MB is located at 664 nm. The absorption intensity decreases with time under UV light irradiation for all the samples, corresponding to the degradation of MB. Figure 5d plots the photocatalytic degradation curves for all the samples. Both pure P25 and Co_3_O_4_-coated P25 powders can degrade MB under UV light. Meanwhile, almost no degradation of MB is observed in UV light without catalyst, demonstrating that MB is stable under UV light. However, 100- or 200-cycle Co_3_O_4_-coated P25 powders show much higher photocatalytic activity compared to pure P25 powders. The degradation efficiency of Co_3_O_4_-coated P25 can reach nearly 100% in 1.5 h, while that of the pure P25 is only about 80%.

The recycling tests were also carried out to determine the stability of the composite catalysts of Co_3_O_4_-coated P25 powders. No decay of photocatalytic efficiency is observed in 200-cycle Co_3_O_4_-coated P25 samples after repeatedly used in MB photodegradation for three times.

**Fig. 6:**
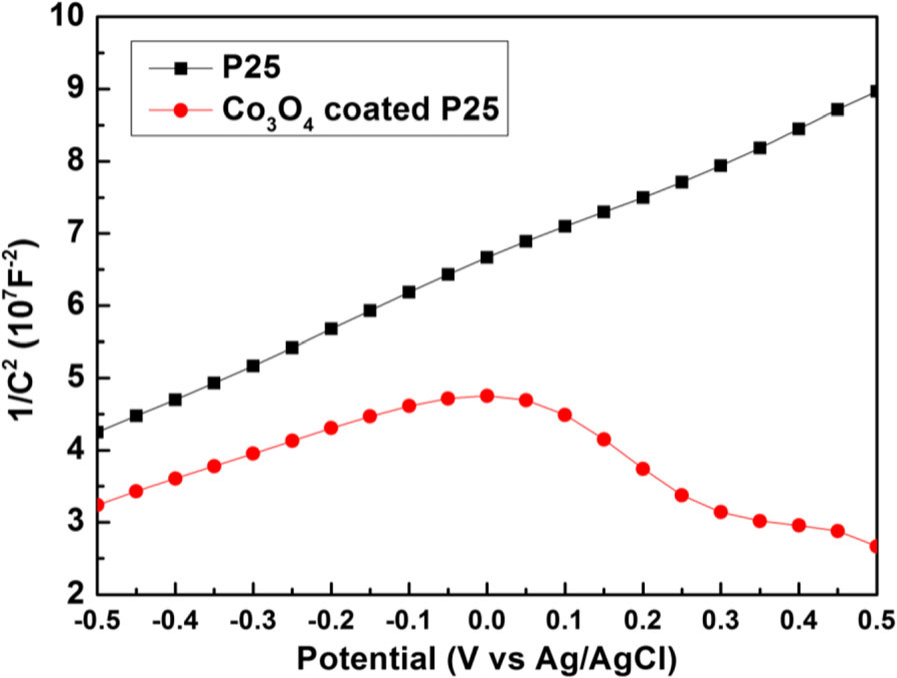
Mott-Schottky plots of pure P25 and Co_3_O_4_-coated P25 in 1 M NaOH aqueous solution with frequencies of 1 and 2 kHz in the dark

The enhanced photocatalytic activity of Co_3_O_4_-coated P25 powders could be attributed to the formation of p-n junction between Co_3_O_4_ and TiO_2_. Figure 6 records Mott-Schottky plots of P25 with or without 200-cycle Co_3_O_4_ coating. Pure P25 samples exhibit the Mott-Schottky plot with positive slope, suggesting the n-type semiconductor with electron carriers. The Mott-Schottky plot with negative slope implies the p-type semiconductor with hole carriers. For 200-cycle Co_3_O_4_-coated P25 catalyst, the co-existence of positive and negative slopes with similar values in the Mott-Schottky plot can be simultaneously observed, indicating the formation of the p-n junction in our samples. This will help in the separation of photogenerated electron-hole pairs [18, 22, 47].

**Fig. 7:**
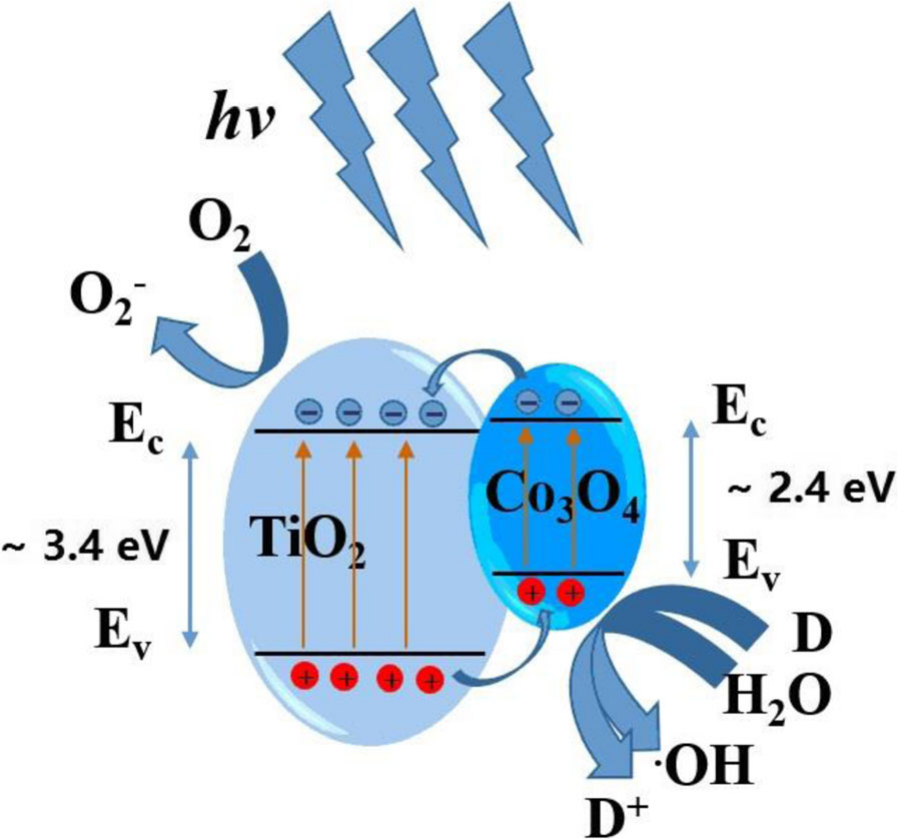
Schematic of energy level and electron-hole movement in Co_3_O_4_–TiO_2_ p-n junction structure under UV light irradiation

Figure 7 illustrates the schematic of energy level and electron-hole movement in Co_3_O_4_–TiO_2_ p-n junction structure. Co_3_O_4_ exhibits a much smaller band gap (~2.4 eV) than TiO_2_ (~3.4 eV). Upon ultraviolet light irradiation, electron-hole pairs can be generated in both Co_3_O_4_ and TiO_2_. According to the energy level structure in Fig. 7, photogenerated electrons would move from conduction band of Co_3_O_4_ to that of TiO_2_. In contrast, holes are injected from the valence band of TiO_2_ to that of Co_3_O_4_. As a result, a high concentration of electrons and holes are formed in the conduction band of TiO_2_ and valence band of Co_3_O_4_, respectively. The electron-hole pair recombination is effectively hindered due to the separation of photogenerated electrons and holes. The separated electrons and holes are then free to undergo reactions with the reactants adsorbed on the photocatalyst surface and enhance the photocatalytic activity. Therefore, the Co_3_O_4_–TiO_2_ p-n junction structure exhibits the better photocatalytic property than pure TiO_2_.

**Fig. 8:**
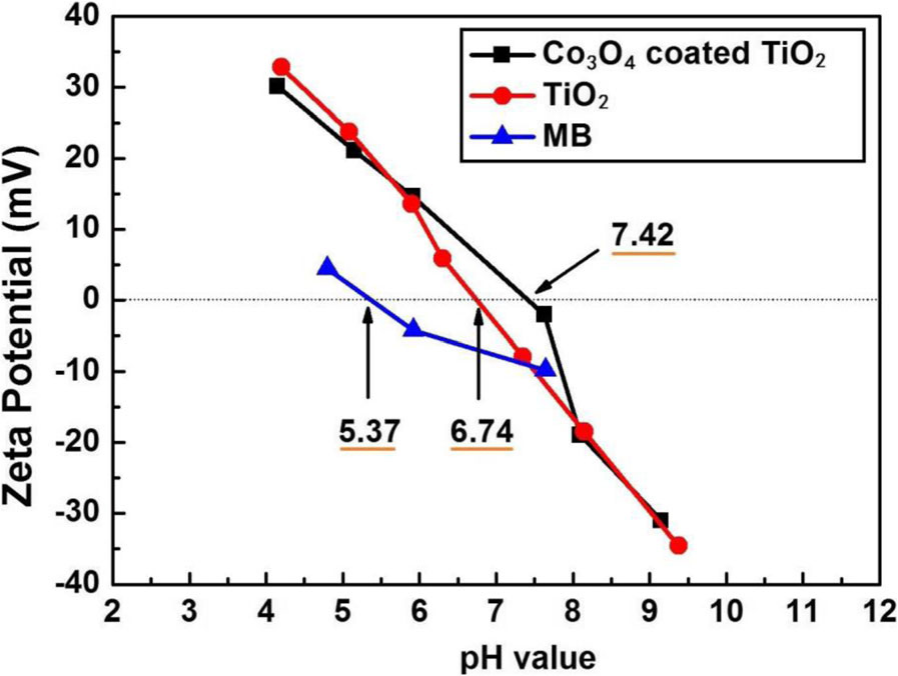
Zeta potential of MB, P25, and Co_3_O_4_-coated P25 in aqueous solutions as a function of pH values

In addition, in order to evaluate the influence of the isoelectric point (IEP) on the absorption of MB, the IEP was detected using the Zeta potential measurement, as shown in Fig. 8. The IEP of MB, pure P25, and 200-cycle Co_3_O_4_-coated P25 in aqueous solutions are determined to be 5.37, 6.74, and 7.42, respectively. The pH value of MB dye and P25 or Co_3_O_4_-coated P25 aqueous suspension is measured to be 6.68. According to the IEP results, the MB dye carries net negative charge while both catalysts carry positive charge. Moreover, Co_3_O_4_-coated P25 powders have more positive charges than pure P25. Therefore, the Co_3_O_4_ coating could promote the adsorption of MB on P25, which is beneficial to the enhancement of photocatalytic activity.

**Fig. 9:**
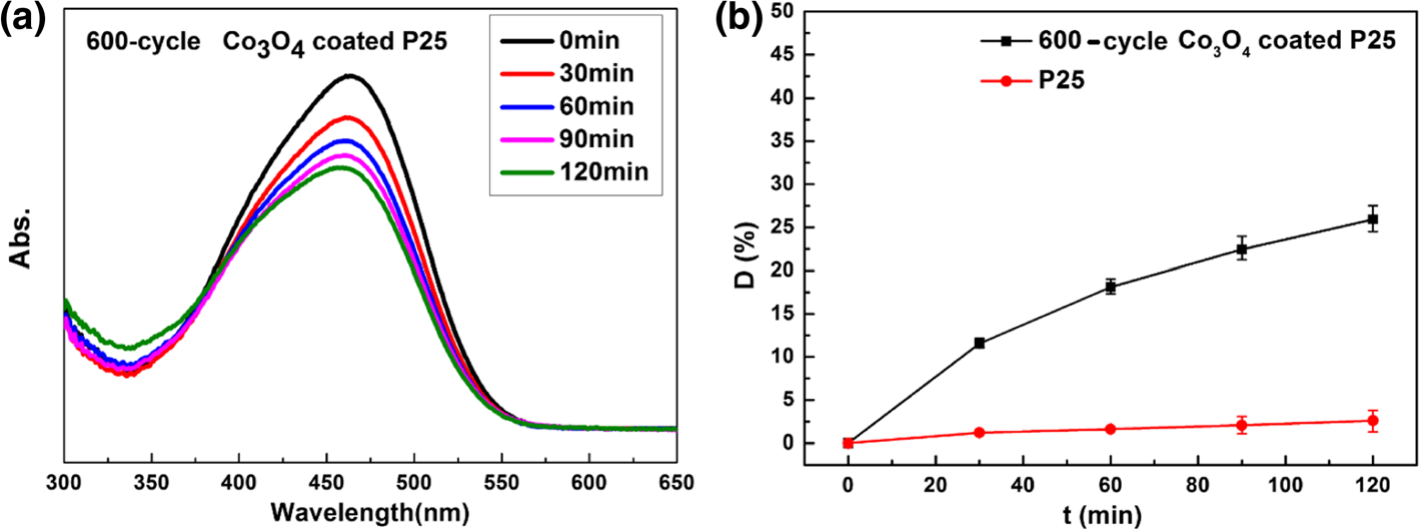
Visible photocatalytic activity of 600-cycle Co_3_O_4_-coated P25 powders. **a** UV-visible absorption and **b** degradation curves of MO solution under visible light irradiation

Finally, the photo-degradation test of methyl orange (MO) using Co_3_O_4_-coated P25 powders was also conducted under visible light illumination. The 200-cycle Co_3_O_4_-coated P25 sample shows no photocatalytic activity for degradation of MO. It can be ascribed to the fact that there exists only trace Co_3_O_4_ on the P25 surface. The trace Co_3_O_4_ cannot absorb enough visible light to stimulate the catalytic reactions. Therefore, we prepared 600-cycle Co_3_O_4_-coated P25 sample by thermal ALD to introduce more Co_3_O_4_ nanoparticles onto P25 powders. The photocatalytic activity in decomposition of MO dye has been examined under visible light irradiation (*λ* ≥ 420 nm), as recorded in Fig. 9. Six hundred-cycle Co_3_O_4_-coated P25 shows visible photocatalytic activity with degradation of ~ 26% MO in 120 min. This can be explained by considering the Co_3_O_4_ nanoparticles activity under visible light due to its narrow bandgap (~ 2.4 eV) as confirmed by Fig. 4d [28].

## Conclusions

In summary, Co_3_O_4_-coated P25 p-n junction powder photocatalysts have been successfully prepared by PEALD. The structure, morphology, composition, and bandgap of these modified P25 powders have been characterized systematically. The photocatalytic activity of MB degradation under UV light has been explored deeply. The anatase structure and crystallite size of P25 powders do not change after 100- and 200-cycle Co_3_O_4_ deposition. However, under UV light, the Co_3_O_4_-coated P25 powders exhibit the degradation rate of almost 100% in 1.5 h. The UV photocatalytic activity has been evidently enhanced compared with pure P25 powders. The Mott-Schottky plots of photocatalyst powders confirm the p-n heterojunction formation in Co_3_O_4_–TiO_2_ nanocomposite materials, which is beneficial to the separation of photogenerated electron-hole pairs. In addition, the IEP results also indicate that the Co_3_O_4_ coating could promote the adsorption of organic dyes of methylene blue on P25 powders. Above all, ALD is a promising and powerful technology to construct effective p-n junction photocatalyst via surface modification.
